# A Startling Showing

**Published:** 1903-12

**Authors:** 


### A STARTLING SHOWING.

According to Dr. M. L. Graves, Superintendent of the Southwestern Insane Asylum at San Antonio, the increase of insanity in Texas is most startling. Dr. Graves says:

"Nineteen years ago the insane population of Texas, as far as the records of the asylum show, was 450. The present population is more than 3200, while the United States census reports show the increase in our total population for the last two decades to be 1,456,961. These interesting figures are fraught with great importance to the race. They show that our insane population has increased more than sevenfold in less than twenty years, while the general population has not quite doubled in the same time."

It would be interesting to know the causes that have resulted in this unprecedented growth of insanity. It would be more so to institute measures to check it. Let me see; at that rate, in nineteen years more, we should have a population of 6,000,000, and 22,400 insane. Make your own calculation how long it will require for everybody in Texas to become insane! Taking the present population at 3,000,000, in round numbers, and Dr. Graves' figures in round numbers, there was one insane person to every 3333 people in Texas, nineteen years ago (in 1884). At present there is one to every 937. Nineteen years hence (1922), there will be one in every 267, and in nineteen years more—or thirty-eight years hence, say in 1941—there will be one insane person to every 76 people, big and little. That is to say, in 1941 there will be 12,000,000 people, and 156,800 insane. Our three asylums have an aggregate capacity of 3200. It will require one hundred and fifty asylums of the same capacity to care for them in 1941. These figures should be pondered by our law makers who see no reason for a record of vital statistics, and the application of the principles of sanitary science. They are respectfully commended to the attention of Hon. Travis Henderson, and other honorable Senators.

---

THE AMERICAN-INTERNATIONAL CONGRESS ON TUBERCULOSIS will meet in St. Louis October 3, 4 and 5, 1904. This is the outgrowth of the American Congress on Tuberculosis which was organized by the Medico-Legal Society of New York in 1900, mainly through the efforts of that grand man, Judge Clark Bell, long time president of that society, and now and for many years editor and publisher of the *Medico Legal Journal.* The American Congress on Tuberculosis held meetings in 1900, 1901, 1902 and 1903. In 1902 there was a split off of some dissatisfied delegates and officers, because Judge Bell, a layman, had made the mistake of inviting certain doctors who were objectionable to the New York doctors from an ethical standpoint. They "bolted," and carried with them a few delegates and the secretary, one Brown of Georgia. The bolters then proceeded to organize, and taking the name under which the American Congress on Tuberculosis is chartered, procured a charter of their own, and, of course, as is usually the case in such breaks, they began at once to villify Clark Bell and the original congress. They announce that they are *it,* and will hold a meeting in Washington in 1905, "after the deluge." Well, let them. Nobody objects. This concern bears the same relation to the American Congress on Tuberculosis that George Clark's bolt bore to the State Democratic convention in 1896, and Cleveland's Buckner-Morrison convention bore to the Democratic party. Meantime the original American Congress on Tuberculosis, having the recognition, backing, support, co-operation and authority of the State Department of the United States—Mr. Hay himself being first Honorary President, and that of the World's Fair management—was accorded a position on the program of the World's Fair Congresses to be held in St. Louis next year. It has grown to such proportions, attracting attention of sanitarians, lay and professional, in foreign countries—many of which have already appointed delegates—that, upon suggestion of the management of the World's Fair Congresses, it is expanded into an International Congress, which is now being organized by the executive committee of the original congress. Its success is assured. A widespread interest, amounting to enthusiasm, prevails, and already most of the States have appointed delegates. It must be borne in mind that the problem of preventing the spread of consumption is not alone a medical question; it is medico-legal, and we need and must have the co-operation and assistance of governors, congressmen, statesmen, legislators, lawyers, engineers, educators, railroad men and all who are interested in the integrity and welfare of the race; because, doctors, alone, can not secure the legislation necessary to vitalize any such reform movement. The International Congress on Tuberculosis at St. Louis next October is going to inaugurate a warfare on the great white plague which will result in inestimable good to the world. I hope all my readers will interest themselves in the campaign.

